# Contrast in Mycorrhizal Associations Leads to Divergent Rhizosphere Metabolomes and Plant–Soil Feedback Among Grassland Species

**DOI:** 10.1111/ele.70318

**Published:** 2026-02-11

**Authors:** Marina Semchenko, Pierre Pétriacq, Sylvain Prigent, Sirgi Saar, Greete Horn, John Davison, Kadri Koorem, Mari Moora, Kristjan Zobel

**Affiliations:** ^1^ Institute of Ecology and Earth Sciences, University of Tartu Tartu Estonia; ^2^ Department of Earth and Environmental Sciences University of Manchester Manchester UK; ^3^ UMR1332 BFP University of Bordeaux, INRAE Villenave d’Ornon France; ^4^ Bordeaux Metabolome, MetaboHUB, PHENOME‐EMPHASIS Villenave d'Ornon France

**Keywords:** arbuscular mycorrhizal fungi, generalist fungi, host specificity, plant–soil feedback, rhizosphere metabolome, root exudates, symbiosis regulation

## Abstract

Species‐specific feedback between plants and soil microbial communities is an important driver of vegetation dynamics. Arbuscular mycorrhizal (AM) fungi colonise most terrestrial plants but are not expected to generate specific feedbacks due to low host specificity. We tested whether variation in mycorrhizal associations and associated rhizosphere metabolomes among co‐existing temperate grassland species leads to species‐specific plant–soil feedback. More mycorrhizal plant species showed more divergent plant–soil feedback: they experienced reduced growth and mycorrhizal colonisation in soils originating from weakly mycorrhizal species, but feedback became neutral in soil from species with similar mycorrhizal strategies. The species with the most self‐promoting soil feedback was characterised by strong metabolome shifts related to stress and immune responses following soil inoculum manipulation, while the metabolomes of species with more negative feedback were unresponsive. This study demonstrates that AM fungi can generate species‐specific plant–soil feedback, which can be predicted from plant mycorrhizal strategies and rhizosphere chemistry.

## Graphical abstract

Most terrestrial plants are colonised by arbuscular mycorrhizal fungi but vary in the degree to which they benefit from and depend on these fungi. Here we show that plants can make the interaction with mycorrhizal fungi more beneficial to themselves by regulating the chemical composition of organic compounds released by plant roots into the soil.

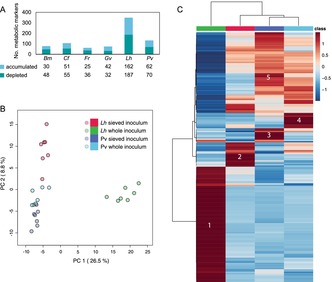

## 1 Introduction

Plant–soil feedback (PSF), where plants cause changes in soil properties that in turn affect subsequent plant growth and performance, plays an important role in plant community dynamics and ecosystem functioning (Bever 2003; Mangan et al. 2010; Maron et al. 2011; Schnitzer et al. 2011; Bennett et al. 2017; Thakur et al. 2021). Many plants exhibit PSF that is species‐specific—individuals grow better or worse in soil previously occupied by conspecifics compared to other species, reflecting positive or negative species‐specific PSF, respectively. Such species‐specificity is often attributed to host‐specificity in plant‐microbial associations. In particular, negative plant–soil feedback is often attributed to host‐specific pathogenic fungi or oomycetes (van der Putten et al. 1993; Klironomos 2002; Maron et al. 2011; Domínguez‐Begines et al. 2021; Wang et al. 2023), while mycorrhizal fungi are expected to make feedbacks more positive (Callaway et al. 2004; Suding et al. 2013; Semchenko et al. 2018; Crawford et al. 2019).

Although species‐specific PSF is frequently observed in experimental studies, the host‐specificity of microbial taxa that mediate PSF is rarely quantified (Mommer et al. 2018). Available data on fungal and oomycete pathogens shows low levels of host‐specificity (Semchenko et al. 2022). The most widespread mycorrhizal fungi—arbuscular mycorrhizal (AM) fungi—are obligate biotrophs, and their diversity is relatively low compared with the vast diversity of plant hosts that they colonise. These factors limit the potential for high host‐specificity in plant‐AM fungal associations, which is also supported by empirical studies (Davison et al. 2015; Sepp et al. 2019; Kivlin et al. 2022). It can, therefore, be expected that the potential for AM fungi to generate species‐specific PSF and thereby modulate species co‐existence is rather limited. Yet, some case studies show that AM fungi can generate not only positive non‐specific PSF but also negative and species‐specific PSF (Bever 2002; Bennett and Bever 2009). This raises the question about the potential alternative mechanisms by which interactions with generalist mycorrhizal fungi can lead to specific PSF.

Although AM fungi are characterised by wide host ranges, plant hosts exhibit broad variation in their reliance on AM symbiosis (Chaudhary et al. 2016; Romero et al. 2023). This can be seen both in terms of the growth response to AM fungal colonisation, which can vary from strongly positive to negative and is often context‐dependent (Hoeksema et al. 2010; Johnson et al. 2015), as well as in terms of the extent of mycorrhizal colonisation—some plant species are always heavily colonised by AM fungi, while others are colonised sparsely (Hempel et al. 2013; Moora 2014). It is unknown how such variation in the extent of mycorrhizal associations relates to specificity in plant‐mycorrhizal interactions and whether there are predictable links between species‐specific PSF and different aspects of mycorrhizal associations.

We predict that there are two potentially interconnected ways in which AM fungi can generate species‐specific PSF. Firstly, species‐specific PSF can arise if the strength of mycorrhizal associations varies widely among co‐existing plant species. A strongly mycorrhizal plant species might experience positive feedback in conspecific soil if heterospecific soils belong to non‐mycorrhizal species that locally exhaust the mycorrhizal fungal inoculum pool (Vierheilig et al. 1995, 2000). Similarly, non‐mycorrhizal plants might experience positive PSF when heterospecific soils are occupied by highly mycorrhizal plants: non‐mycorrhizal plants could face higher pressure from mycorrhizal fungi attempting to colonise their roots in soils previously occupied by highly mycorrhizal plants, which could incur a defence cost to plants at the expense of growth (Francis and Read 1995; Wang et al. 2022). Hence, we may expect to observe a range of PSF with neutral PSF between plant species with similar mycorrhizal association strength and more positive PSF with increasing contrast in strategies between co‐existing plant hosts.

Secondly, in addition to the average strength of mycorrhizal association, plant species may also vary in the flexibility of mycorrhizal associations (Zobel et al. 2024; Moora et al. 2025). Species‐specific PSF may be generated among plant species with differential ability to control symbiosis with mycorrhizal fungi (Semchenko et al. 2022). The mechanisms by which plants may regulate AM symbiosis are still poorly understood, particularly in the context of interspecific differences. Limited evidence supports the ability of some model plant species to differentially allocate more carbon towards more beneficial AM fungal species under highly controlled conditions (Bever et al. 2009; Kiers et al. 2011; Ji and Bever 2016). It has also been demonstrated that the exudation of certain compounds, such as strigolactones and flavonoids, plays key roles in the different stages of AM symbiosis establishment (Akiyama et al. 2005; López‐Ráez et al. 2008; Sasse et al. 2018; Tian et al. 2021). Furthermore, evidence is emerging that AM fungi with mutualistic versus parasitic impact on plant performance can trigger differential changes in the root metabolome (Kaur et al. 2022). It is therefore possible that more positive feedback with AM fungi is associated with the ability of some species to modulate rhizosphere metabolome (i.e., soluble organic compounds in the vicinity of plant roots) and achieve a stronger control over AM symbiosis.

Here we examined the capacity of AM fungi to generate species‐specific PSF in a range of co‐existing grassland species with different levels of reliance on mycorrhizal associations. We further linked variation in mycorrhizal associations to species‐specific changes in rhizosphere metabolomes as well as soil nutrient and carbon availability. We predicted that (a) PSF will be more positive among species with more contrasting mycorrhizal association strengths; (b) the presence of AM fungi leads to divergent changes in rhizosphere chemistry among species with different mycorrhizal association strengths; (c) species‐specific PSF can be linked to distinct changes in rhizosphere metabolome. To test these predictions, ten grassland species were exposed to soil inoculum either containing or excluding AM fungi, and the strength of mycorrhizal associations as well as the impact of plant species on rhizosphere chemistry were assessed. In the feedback stage of the experiment, a new generation of plants was grown in conspecific soils and a range of soils previously occupied by other species to assess how PSF varied as a function of mycorrhizal associations.

## 2 Materials and Methods

### 2.1 Study System

Seeds of ten common temperate grassland species were collected from two grasslands on the coast of the Baltic Sea, Estonia: *Briza media* L. (graminoid), *Carex flacca* L. (graminoid), *Leontodon hispidus* L. (forb), *Prunella vulgaris* L. (forb), and *Succisa pratensis* Moench (forb) from Laelatu wooded meadow (58°35′06″ N 23°34′09″ E), and *Carlina vulgaris* L. (forb), *Festuca rubra* L. (graminoid), *Galium verum* L. (forb), *Pimpinella saxifraga* L. (forb), and *Silene vulgaris* (Moench) Garcke (forb) from Uisu alvar grassland (58°38′31″ N 23°30′51″ E). The soil at both sites is rendzic leptosol with neutral pH (soil thickness 5–30 cm) over Silurian limestone bedrock with calcareous moraine (Sepp and Rooma 1970). The grasslands are characterised by low productivity and high plant species richness (Kull and Zobel 1991; Zobel and Liira 1997; Aavik et al. 2008). Soil was collected from each site and used as inoculum for species originating from the corresponding study site.

### 2.2 Soil Conditioning Phase

Sand and soil of the same type (rendzic leptosol) as at the study sites was sterilised using gamma irradiation (25 Gy) and a background soil mixture was prepared by mixing 1 part of soil with 2 parts of sand. Pots were filled with 1.4 kg of soil mixture. To create soil treatments with and without AM fungal presence, 300 g of sterilised or live soil inoculum were added to pots. To homogenise other microbiota across all pots, a liquid microbial wash was prepared by mixing 5 L of water with 1 kg of live soil, which was filtered through a sieve with 32 μm aperture to exclude AM fungal spores and was added to all pots at the rate of 60 mL per pot. Therefore, the treatment with sterilised inoculum and sieved microbial wash lacked AM fungal spores but included soil biota smaller than 32 μm. The sieving approach is efficient in removing AM fungi from soil inoculum due to the large size of their spores (Klironomos 2002; Frew et al. 2025), but it can also reduce the diversity of other free‐living fungi but not bacteria due to dilution effects (Wagg et al. 2014, 2019). In our study, plant responses to inoculum manipulation reflected interactions with AM fungi as (i) whole soil inoculum triggered more divergent PSF compared with the sieved treatment only in highly mycorrhizal species, (ii) divergent responses to different soil legacies could be explained by the mycorrhizal strategy of the species that conditioned the soil, and (iii) growth responses to soil legacies were correlated with changes in mycorrhizal colonisation rates, providing a direct link between plant–soil feedback and AM fungi.

Seeds were surface‐sterilised with sodium hypochlorite, sown onto sterile sand and kept in a greenhouse with additional lighting of 350 μmol/m^2^/s, air at 20°C and a 16 h:8 h day: night cycle. Three weeks later, seedlings were transplanted into pots, each pot included three conspecific seedlings, and each species was represented by seven replicates in each soil treatment (140 pots in total; Figure S1). Plants were grown under conditions specified for seed germination for 2 months (April–June). Pots were kept moist by regular watering, placed randomly on benches and re‐randomised every 2 weeks.

After 6 weeks of growth, plants were well established, and roots were spread throughout the pot volume in six study species ( *B. media* , *C. flacca* , *L. hispidus* , *P. vulgaris* , *F. rubra* , *G. verum* ). We therefore treated soil contained within a pot as rhizosphere soil and collected leachates to assess differences in rhizosphere chemistry. The pots were placed over a plastic container and 250 mL of tap water was added to each pot, resulting in ca 75 mL of leachate collected per pot. The leachates were filtered with syringe filters (pore size 0.2 μm, Minisart, Sartorius Stedim biotech, Göttingen, Germany) and were immediately frozen at −20°C. Leachates were collected four times during 2 weeks and pooled per pot before analysis. Leachates were analysed for total organic C (TOC) using a 5000A TOC‐L analyser (Shimadzu, Japan) and ammonium, nitrate, phosphate and total organic nitrogen (TON) were measured in an Auto Analyser AA3 (Seal Analytical, UK). Subsamples for metabolomics were freeze‐dried, and 10 mg (dry weight) were analysed using UPLC‐QTOF‐MS (LCMS), as previously described (Pétriacq et al. 2017); see further details in the Supporting Information S1.

After 8 weeks of growth, plant shoots were clipped at the soil surface, dried for 48 h at 75°C and weighed. To assess root biomass and AM fungal colonisation, soil cores (diameter of 2.5 cm) were taken from the centre of each pot. Roots were washed, dried at 40°C for 48 h, weighed, and total root mass per pot was calculated based on total soil volume. Root AM fungal colonisation rates were assessed with the trypan blue staining technique as described in Supporting Information S1 (McGonigle et al. 1990). The remaining soil was left undisturbed and stored at 4°C.

### 2.3 Feedback Phase

The conditioned soil and roots from each pot were mixed with an equal weight of sterilised background soil mixture (same as used in the conditioning phase) and used to fill five new pots of 0.5 L volume. Seeds were germinated as described above, and one seedling was planted in the centre of the pot filled with either conspecific soil or four soils conditioned by other species from the same site (Figure S1). Each seedling‐soil combination was replicated five times within each inoculation treatment, resulting in 50 pots per species in total. Due to insufficient seed germination in *S. pratensis* and *P. saxifraga* , these species were excluded from the experiment, resulting in a total of 400 pots distributed among eight focal species. Plants were grown under the same conditions as in the conditioning phase. Aboveground biomass was harvested 8 weeks after planting and dried at 75°C for 48 h. Roots were separated from soil, washed and dried at 40°C for 72 h. Root subsamples were analysed for root AM fungal colonisation as described above.

### 2.4 Statistical Analysis

#### 2.4.1 Conditioning Phase

A general linear model was used to assess the effect of the soil inoculation treatment (whole vs sieved), plant species identity and their interaction on plant total biomass. To describe variation in species mycorrhizal strategy, a general linear model was used with logit‐transformed proportion of root AM fungal colonisation in whole soil inoculum treatment as a dependent variable and plant species identity as a fixed factor. In the following analyses, the mean percentage of mycorrhizal colonisation in the whole soil treatment was used as a species‐level, continuous trait that describes the strength of mycorrhizal association.

To describe the interactive effect of inoculation and mycorrhizal strategy on soil chemical properties, redundancy analysis was conducted on the five measured chemical traits (DOC, DON, nitrate, ammonium, and phosphate concentrations; scaled and ln‐transformed) with the inoculation treatment, strength of mycorrhizal association, and their interaction as predictor variables. The analysis was based on 999 permutations, which involved permuting species as blocks. The analysis was performed using the *rda* function within the vegan package (Oksanen et al. 2020) and the *how* function within the permute package (Simpson 2022). The data were visualised with a PCA plot and the *envfit* function from the vegan package.

#### 2.4.2 Metabolomics

Detected LCMS features were normalised (median normalisation, cube root transformation, and Pareto scaling) and explored using Principal Component and Clustering analyses and Volcano plots with MetaboAnalyst (v 5) and redundancy analysis and permutations as described above for chemical properties. Chemodiversity was calculated as Shannon diversity and analysed in a linear mixed model with mycorrhizal association strength and soil inoculation (and their interaction) as fixed factors and focal species identity as a random factor. In order to test the capacity of the soil metabolome to predict AM fungal interactions among species, we deployed predictive metabolomics (Dussarrat et al. 2022, 2023; Dollinger et al. 2023)—generalised linear models were performed using the *caret* package (Kuhn 2008) for two categorical traits (inoculation treatment and the presence/absence of mycorrhizal colonisation; binomial distribution) and one continuous trait (mycorrhizal colonisation percentage; Gaussian distribution) as response variables and metabolomics features as predictor variables. Overall model accuracy was calculated as the proportion of correctly predicted classes (or *R* ^2^ between observed and predicted values for the extent of mycorrhizal colonisation) among all predictions performed in 100 random models. The *p* value was computed using a one‐sided binomial test comparing overall accuracy with the No Information Rate (the expected accuracy for a dummy model that always predicts the most represented class).

#### 2.4.3 Feedback Phase

Plant–soil feedback estimates were obtained from linear models performed for each focal species and each heterospecific soil legacy in either whole soil or sieved inoculum treatments. The models included log‐transformed plant dry biomass as a dependent variable and soil legacy treatment (conspecific or heterospecific soil legacy) as a fixed factor. In total, 64 individual PSF values were calculated (8 species × 2 inoculation treatments × 4 heterospecific soil legacies). As the distribution of PSF estimates did not satisfy the assumptions of normal distribution (based on Shapiro–Wilk test) and equality of variances (Levene's test) between the two inoculation treatments, Wilcox rank sum test was used to test for the inoculation treatment effect. A linear mixed model was performed with PSF estimates from the whole inoculum treatment as a response variable, the main effects and interaction between mycorrhizal association strengths of the focal and heterospecific soil legacy species as fixed predictors, and focal and heterospecific soil species identity as random intercepts. The same modelling approach was used for predicting root mycorrhizal colonisation response instead of biomass PSF.

To examine how biomass production and soil chemical properties at the end of soil conditioning affected PSF, we calculated PSF values (as log‐ratios) based on all pairwise comparisons of plant biomass in conspecific soil (5 replicates) and heterospecific soil (four soils; five replicates each). This resulted in 708 PSF values (800 theoretically but reduced due to mortality). Analogously, log‐ratios of all pairwise differences in chemical properties (C, N, P, chemodiversity) or conditioning phase biomass in corresponding soils were calculated and used in models as predictors of PSF in interaction with inoculation treatment. Focal and heterospecific soil legacy species identity were included as random intercepts, and inoculation treatment as a random slope to allow for variation in PSF‐predictor slopes among species. In addition, the Euclidean distances in metabolomic space between samples were calculated and used as a predictor of PSF strength (i.e., absolute value of PSF) to test if greater differences in rhizosphere metabolomes result in stronger PSF.

All analyses were performed in R version 4.3.3 (Team 2024). Linear mixed models were performed using the lme4 package (Bates et al. 2015), emmeans (Lenth 2025), and the car package (Fox and Weisberg 2019).

## 3 Results

### 3.1 Interspecific Variation in Mycorrhizal Colonisation and Growth Response to Inoculation Treatment

Plants inoculated with sieved microbial inoculum mostly lacked AM fungal colonisation (low levels detected in 15% of samples in the conditioning phase and no colonisation detected in the feedback phase). Plant growth response to soil inoculation varied widely among species, from 27 times higher to 1.5 times lower biomass production in the whole compared with sieved soil treatment (Figure S2; interaction between species and inoculation *F*_9,117_ = 10.1, *p* < 0.001; adjR = 0.77). Within the whole soil inoculation treatment, species differed widely in mean levels of mycorrhizal colonisation (from < 1% to 72%, species identity effect *F*_9,56_ = 17.5, *p* < 0.001; adjR = 0.70), which we hereafter refer to as the strength of mycorrhizal association for a given species.

### 3.2 Plant–Soil Feedback as a Function of Soil Inoculation and Plant Mycorrhizal Strategy

**FIGURE 1:**
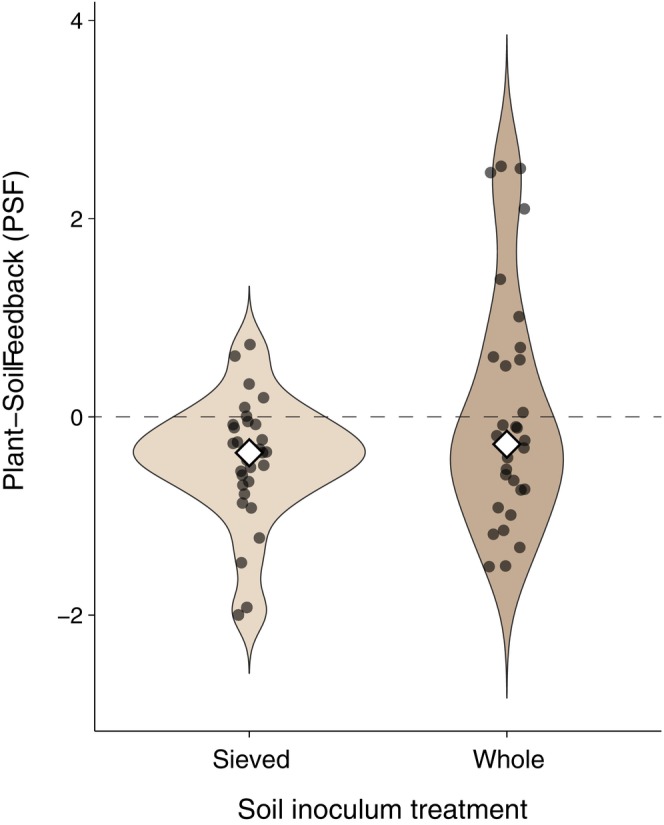
Distributions of plant–soil feedback (PSF) estimates in soil with either sieved inoculum that excluded arbuscular mycorrhizal (AM) fungi or whole soil inoculum. PSF was estimated as the difference in ln‐transformed plant dry mass when grown in soil previously occupied by conspecifics versus a different species. Eight focal plant species were grown in conspecific soil and four soils conditioned by other species, with sieved or whole inoculum, resulting in 64 PSF values in total. Positive and negative PSF values indicate increased or reduced plant growth in conspecific compared with heterospecific soil, respectively. Open symbols show median values.

**FIGURE 2:**
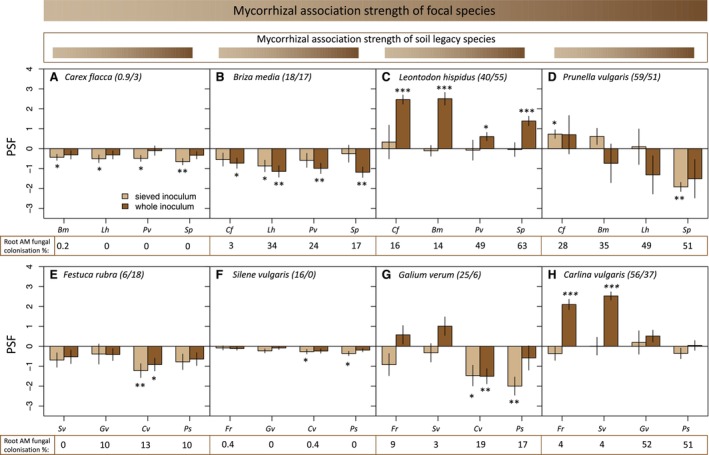
Plant–soil feedback is influenced by the inoculation treatment and depends on the difference in mycorrhizal association strength between successive plant species occupying the soil. Eight focal plant species (A–H) were each exposed to conspecific soil legacy and four heterospecific soils conditioned by different plant species. Positive and negative plant–soil feedback (PSF) values indicate increased or reduced plant growth in conspecific compared with heterospecific soil legacies, respectively, on log_e_ scale. Soils were initially inoculated with either whole soil, including arbuscular mycorrhizal fungi, or sieved soil inoculum that excluded AM fungi (sieve aperture 32 μm). Plant species and soil legacies of different species are arranged in the order of increasing mean root mycorrhizal colonisation % in the whole soil inoculation treatment during the soil conditioning stage (from A to D for species from Site 1 and E‐H for Site 2; % colonisation indicated with the first number in parentheses next to focal species name). Mean root mycorrhizal colonisation in the feedback stage in the whole soil inoculation treatment is indicated by the second number in parentheses for conspecific soil legacies and beneath species abbreviations for each heterospecific soil legacy. The identity of species that conditioned heterospecific soils is indicated beneath the barplots with abbreviations (*Cf—Carex flacca; Cv—Carlina vulgaris; Bm—Briza media; Fr—Festuca rubra; Gv—Galium verum; Lh—Leontodon hispidus; Ps—Pimpinella saxifraga; Pv—Prunella vulgaris; Sv—Silene vulgaris; Sp—Succisa pratensis*). Error bars indicate standard errors of predicted means. Asterisks indicate significant deviation of PSF from zero (based on a linear model comparing log_e_‐transformed plant biomass in conspecific vs heterospecific soil legacy). *p < 0.05; **p < 0.01; ***p < 0.001.

Mean plant–soil feedback was not significantly affected by the soil inoculation treatment (Wilcoxon test, *p* = 0.307). However, whole soil inoculum generated significantly more divergent PSF among examined plant–soil combinations than when plants were exposed to the sieved soil inoculum (Levene test, *F*_1,62_ = 9.6; *p* = 0.003, Figure 1). In the sieved inoculum treatment, PSF was consistently negative for weakly mycorrhizal species and neutral for highly mycorrhizal species (Table S1, Figure 2). In the whole soil treatment, PSF in species with higher mycorrhizal association strength depended more strongly on the identity of heterospecific soils—the species identity of heterospecific soil inoculum explained from 9% to 89% of variation in plant biomass for species with mean AM root colonisation ranging respectively from 1% to 59% (Table S1). Strongly mycorrhizal species experienced more positive PSF when grown in soil of weakly mycorrhizal species but more neutral PSF when grown in soils of other highly mycorrhizal species (Figure 2).

**FIGURE 3:**
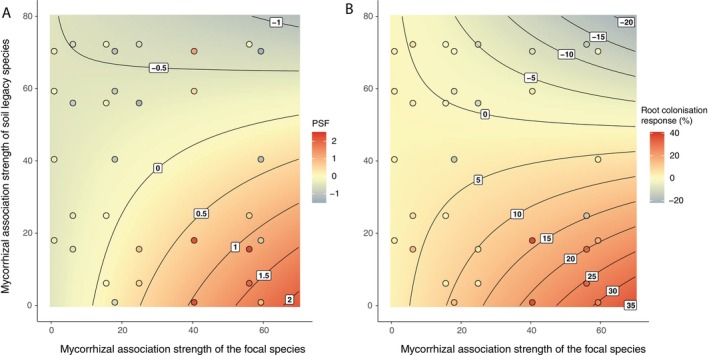
The most positive plant–soil feedback (PSF, panel A) and most positive response in root mycorrhizal colonisation (panel B) is observed when a focal species with strong mycorrhizal association is exposed to a contrasting heterospecific soil legacy left behind by a weakly mycorrhizal species (bottom right corner on both panels). PSF and root colonisation response were calculated as the difference in ln‐transformed dry mass and colonisation % between conspecific and heterospecific soil legacy treatments, respectively. Positive and negative PSF or colonisation response values indicate increased or reduced plant growth or root colonisation in conspecific compared with heterospecific soil legacies, respectively (indicated by colour variation from red to blue, respectively). The colour surface and contour lines represent predicted values, while the colour of data points indicates observed values. Data from the whole inoculum treatment were used in calculations, and significant interactive effects between the mycorrhizal association strengths of focal and soil legacy species were detected in models predicting both biomass PSF and colonisation response (*p* < 0.05).

When mycorrhizal association strengths of focal species and species that conditioned heterospecific soils were used as predictors of PSF in the whole inoculum treatment, significant interactive effects of these two continuous predictors were confirmed (*F*_1,17_ = 12.4, *p* = 0.003). Highly mycorrhizal species experienced positive PSF when exposed to soil conditioned by species with low mycorrhizal colonisation; PSF shifted towards neutral and negative as the contrast in mycorrhizal strategies between focal species and species that conditioned the soil decreased (Figure 3A). Similarly, highly mycorrhizal plants exhibited higher AM fungal colonisation rates in conspecific than in heterospecific soil previously occupied by less mycorrhizal species; AM fungal colonisation rates became more similar between conspecific and heterospecific treatments as the contrast in mycorrhizal strategies between plant species declined (interactive effect of focal and soil conditioning species *F*_1,18_ = 7.7, *p* = 0.013; Figure 3B). Species with low mycorrhizal association strength experienced consistently negative PSF and low AM fungal colonisation rates independent of heterospecific soil identity (Figures 2 and 3).

### 3.3 Rhizosphere C, N and P Response to Soil Inoculation Treatment

**FIGURE 4:**
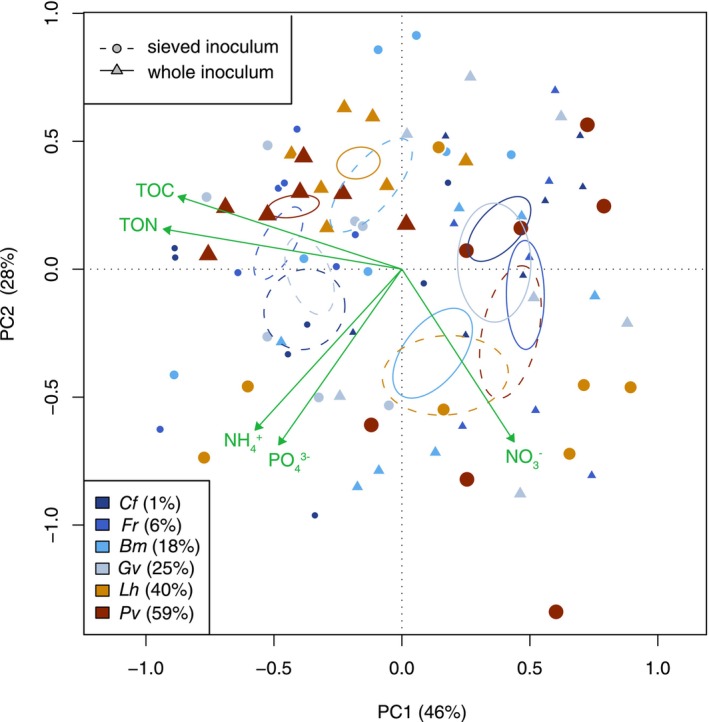
Principal component analysis (PCA) of soil chemical legacies left behind by six grassland species characterised by a range of mycorrhizal association strength. Colour variation from dark blue to brown corresponds to the low to high mean level of arbuscular mycorrhizal (AM) fungal colonisation in the roots of focal plant species (shown in parentheses in %); colonisation rate also scales positively with symbol size. Triangles and circles represent observations from plants that were inoculated with whole soil or sieved inoculum that excluded AM fungi, respectively. Five soil chemical parameters were included in the PCA and their alignment with the PC axes is indicated with green arrows. Ellipses indicate the standard error of point distributions, with solid and dashed lines indicating treatments with whole and sieved inoculum, respectively. Significant interactive effects of soil inoculation treatment and mean AM fungal colonisation rate were detected (PERMANOVA, *p* = 0.001, *R* ^2^ = 0.20). Bm, *Briza media* ; Cf, *Carex flacca* ; Fr, *Festuca rubra* ; Gv, *Galium verum* ; Lh, *Leontodon hispidus* ; Pv, *Prunella vulgaris*.

Plant species with contrasting mycorrhizal association strengths shifted soil chemical composition in opposing directions in response to soil inoculum manipulation (redundancy analysis, interactive effect of inoculation and mycorrhizal association strength, *F*_1,80_ = 21.4, *p* = 0.011, *R* ^2^ = 0.24, Figure 4). Highly mycorrhizal species increased the concentrations of organic carbon and nitrogen in their rhizosphere and reduced nitrate availability in the whole compared with the sieved inoculum treatment; the opposite response to inoculation was observed in species with weak mycorrhizal associations (Figure 4; Table S2).

Variation in PSF was not significantly related to variation in rhizosphere organic C, nitrate and ammonium (Table S3). In the whole soil inoculum treatment, PSF was more positive for plant–soil combinations where concentrations of dissolved organic N were lower in conspecific than in heterospecific soils. In the sieved inoculum treatment, more positive PSF was detected when conspecific soil was higher in phosphate and was characterised by lower biomass production in the conditioning phase than heterospecific soil (Table S3).

### 3.4 Metabolic Signatures of Rhizosphere Soil Solutions

**FIGURE 5:**
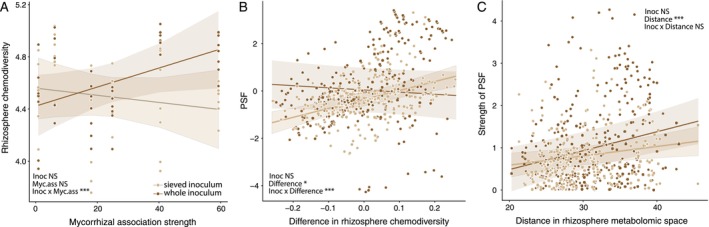
The response of rhizosphere chemodiversity (estimated as Shannon diversity of detected metabolites) to soil inoculation treatment (whole vs. sieved excluding AM fungi) across species varying in mycorrhizal association strength (A), the dependence of plant–soil feedback (PSF) on the difference in chemodiversity between conspecific and heterospecific soil legacies (calculated as log‐ratio with zero indicating equal chemodiversity and positive values indicating higher chemodiversity in conspecific than heterospecific soil, B) and the relationship between the strength of PSF (absolute value of PSF) and metabolomic distance between conspecific and heterospecific soil legacies (C). Lines and shaded areas indicate regression lines and 95% confidence intervals for each soil inoculation treatment. The significance of predictors in linear mixed models: NS—*p* > 0.05, *p < 0.05, ***p < 0.001.

Rhizosphere chemodiversity was significantly higher in whole soil than in sieved inoculum treatment in highly mycorrhizal species but was unaffected by inoculation in weakly mycorrhizal species (interactive effect of inoculation and mycorrhizal association strength, *F*_1,74_ = 12.1, *p* < 0.001, *R* ^2^_m_ = 0.15; Figure 5A). Higher chemodiversity in soils conditioned by conspecifics than heterospecifics resulted in significantly more positive PSF in the sieved inoculum treatment, but such a relationship was absent in the full inoculum treatment (interactive effect of inoculation and difference in chemodiversity on PSF—*F*_1,277_ = 15.7, *p* < 0.001, *R* ^2^_m_ = 0.07, Figure 5B). The composition of rhizosphere metabolomes also differed significantly between species and soil inoculation treatments (redundancy analysis, interactive effect *F*_5,72_ = 2.28, *p* < 0.001, *R* ^2^ = 0.44), and greater differences in metabolome composition resulted in stronger PSF independent of inoculation treatment (i.e., greater contrast in plant growth between soils with contrasting metabolomes; *F*_1,224_ = 12.0, *p* < 0.001, *R* ^2^_m_ = 0.08; Figure 5C).

**FIGURE 6:**
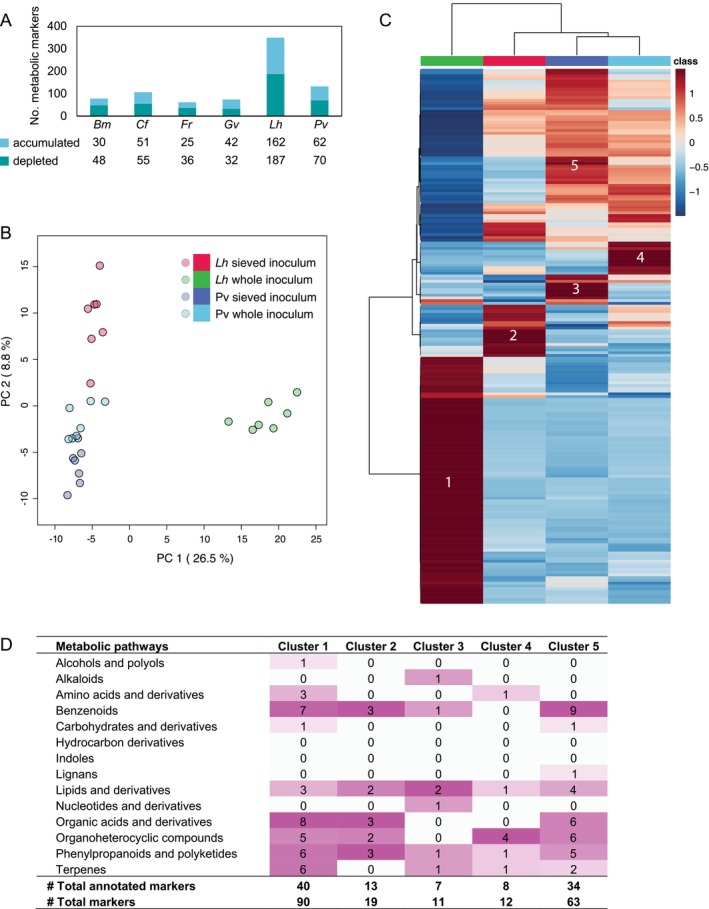
Untargeted metabolic profiles of rhizosphere soil solutions. (A) Numbers of metabolic markers that were accumulated (blue) or depleted (green) in whole soil compared with sieved inoculum excluding AM fungi (based on Volcano plots; fold change > 1.5, *p* < 0.05). (B–D) Metabolic responses of rhizochemicals to soil inoculum treatment (sieved vs whole) in *Leontodon hispidus* (*Lh*) and *Prunella vulgaris* (*Pv*) as two species with similar AM fungal colonisation rates but distinct plant–soil feedback. (B) PCA of 1173 metabolomic features using normalised intensities (median normalisation, cube root transformation and Pareto scaling). (C) Clustering analysis (based on Pearson's correlation and Ward clustering) of 195 metabolic markers that differed significantly between the two species and two soil inoculation treatments (ANOVA, *p* < 0.01 with adjusted false discovery rate correction), showing 5 main clusters. See panel B for colour coding of the species and inoculation treatments. Each row indicates the significant metabolic markers with relative intensity shown as a heatmap (blue, depleted; red, accumulated). (D) Distribution of the markers belonging to each of the five clusters among metabolic pathways based on chemical ontologies.

Rhizosphere metabolome composition and its response to inoculation treatment were not significantly related to species mycorrhizal association strength (redundancy analysis, main effect *F*_1,82_ = 3.2, *p* = 0.746 and interactive effect *F*_1,80_ = 1.4, *p* = 0.546). Instead, highly species‐specific responses were observed. The strongest response to soil inoculum manipulation was detected in *L. hispidus* (Figure S3A). This was confirmed by a clustering analysis of 668 significant metabolic markers (ANOVA, *p* < 0.01, false discovery rate, FDR, corrected; Figure S3B). While the rhizosphere of *L. hispidus* experienced significant changes in 349 metabolic signals in response to the inoculation treatment (178 after FDR correction; fold change > 1.5, *p* < 0.05), other species showed little or no change in rhizosphere metabolite composition (11 for *P. vulgaris* and none for other species under FDR correction; Figure 6A).

In a direct comparison of *L. hispidus* and *P. vulgaris* , species with similar AM fungal colonisation rates but positive and negative PSF, respectively (Figure 2C,D), contrasting metabolomic responses to soil inoculation were detected (Figure 6B). Clustering analysis revealed a large cluster of 90 metabolic markers that were enriched in the *L. hispidus* rhizosphere in the whole inoculum treatment (Figure 6C, cluster 1). This cluster mainly contained metabolites involved in immune and environmental responses, such as phenylpropanoids (coumaric acids, flavonoids, isoflavones and derivatives of the defence hormone salicylic acid and antifungal pyranone) and terpenes (Figure 6D, Table S4). Cluster 1 also included primary metabolites such as amino acid derivatives, organic acids, sugars and lipids. Cluster 2 contained 19 markers that accumulated in *L. hispidus* in the sieved inoculum treatment (Figure 6C), which included phenylpropanoids such as flavonols, phenolic glycosides and defence hormone salicylic acid, together with some lipids (Figure 6D, Table S4). Cluster 3 included secondary compounds (phenolics, alkaloid, terpene) and primary metabolites (nucleoside, nitrophenol) that increased in *P. vulgaris* in the sieved inoculum treatment (Figure 6C,D, Table S4). Cluster 4 included secondary (terpene, flavonol) and primary metabolites (amino acid derivatives, lipids, butyrolactone) that increased in the rhizosphere of *P. vulgaris* inoculated with whole soil (Figure 6C,D, Table S4). Finally, 63 markers were reduced in intensity in *L. hispidus* exposed to whole soil inoculum (Figure 6C, cluster 5). There included metabolites involved in stress responses—phenylpropanoids (coumaric acid, flavonol, tannin, phenolic glycoside), glycoside compounds, pyranones, pyrroloindoles and terpenes—and primary compounds such as organic acids, lipids and tryptophan (Figure 6D; Table S4).

Rhizosphere metabolome accurately predicted the soil inoculation treatment and whether a plant was colonised by mycorrhizal fungi (Figure S4). Predictive accuracy for the level of mycorrhizal colonisation (expressed as percentage) was lower than that for the presence of colonisation, but all predictive models were highly statistically significant (Figure S4, *p* < 0.001). The top metabolic predictors (defined as those with a prevalence of over 60% in 100 models performed for each predicted trait) belonged to stress‐related metabolites such as phenylpropanoids, terpenes, and organoheterocyclic compounds, alongside primary metabolites (predominantly amino acids, benzenoids and lipids, Figure S4, Table S5).

## 4 Discussion

Here we demonstrate that the presence of AM fungi in soil does not result in more positive plant–soil feedback (PSF), but feedback becomes more divergent among co‐existing grassland plant species. Divergent PSF was generated by differences in the strength of mycorrhizal associations among co‐existing plant species.

Highly mycorrhizal species were sensitive to the soil conditioned by other species, with more positive PSF observed when soils were conditioned by species with a contrasting mycorrhizal strategy, that is, non‐mycorrhizal or weakly mycorrhizal species. Such positive feedbacks were accompanied by lower rates of root mycorrhizal colonisation in heterospecific soils, suggesting that weakly mycorrhizal species reduce the abundance of AM fungal diaspores in soil and thereby limit recruitment of AM fungi in the roots of more mycorrhizal species (Kabir et al. 1996; Fontenla et al. 1999; Lambers and Teste 2013). On the other hand, species with weaker mycorrhizal associations experienced neutral to negative PSF, which was largely independent of the mycorrhizal strategy of the soil legacy species.

In the absence of AM fungi, PSF could be partially predicted by rhizosphere chemodiversity, with higher chemodiversity in conspecific soil associating with more positive PSF. Similar positive effects of chemodiversity on soil microbial functioning and plant nutrition have been demonstrated in previous studies and were linked to beneficial effects of intercropping in agriculture (Li et al. 2022; Jiang et al. 2024). In the presence of AM fungi, the link between rhizosphere metabolome and PSF was not related to species mycorrhizal association strength, but seemed to be species‐specific: species with similar levels of mycorrhizal colonization showed contrasting PSF patterns and metabolic responses to inoculation. In particular, the species with the strongest positive PSF responded to soil inoculum manipulation with a major shift in metabolites associated with plant defense and stress resistance, while such changes were less pronounced in other species. These findings suggest that AM fungi can play a major role in modifying plant–soil feedback and underlying mechanisms operate at two distinct levels: broad variation in PSF is driven by the contrast in overall mycorrhizal association strength among co‐occurring species, while additional species‐specific variation in PSF may be related to the ability of some species to regulate rhizosphere metabolome in response to mycorrhizal colonization.

### 4.1 Can Rhizosphere Metabolome Shed Light on the Regulation of Mycorrhizal Symbiosis?

We found that the rhizosphere metabolome can be used to successfully predict the presence and extent of root mycorrhizal colonisation across a range of grassland plant species, suggesting that the overall extent of mycorrhizal associations is related to general metabolic markers that may be compared across species. These metabolites broadly correspond to groups known to increase within plant tissues in the presence of AM fungi—plant hormones, secondary metabolites related to defence, and primary metabolites involved in sugar, amino acid and organic acid synthesis (Schweiger et al. 2014; Hill et al. 2018; Kaur and Suseela 2020; Rivero et al. 2021). The strength of PSF could also be predicted from the metabolomic distance between conspecific and heterospecific soils. In addition to compositional changes in the metabolome, we detected consistent changes in the availability of dissolved organic carbon and nitrogen and soil nitrate depletion that were dependent on plant species mycorrhizal strategy: highly mycorrhizal species responded to inoculation with whole versus sieved soil that effectively excluded AM fungi with an increased flow of organic carbon and nitrogen into the rhizosphere and nitrate depletion, while weakly mycorrhizal species responded to inoculation in the opposite way. This suggests that AM fungi can modify soil C and N supply and presumably competition for inorganic N among species with divergent mycorrhizal strategies, which may steer vegetation succession and species co‐existence (Veresoglou et al. 2018; Koziol and Bever 2019).

The species with the strongest positive PSF showed a clear shift in its rhizosphere metabolome in response to whole soil community inoculation, while other species showed weak responses to inoculum manipulation. The metabolic response included modifications in metabolites primarily involved in stress responses, including the defense hormone salicylic acid and its derivatives, a range of phenylpropanoids known to have antimicrobial and antioxidant functions and indicative of general defense priming, as well as terpenes and primary metabolites such as organic acids, which can modify soil P availability (Kaur and Suseela 2020; Stassen et al. 2021; Daryanavard et al. 2023). Previous studies with sorghum and tomato showed significant shifts in the root metabolome in response to different AM fungal species (Rivero et al. 2015). Moreover, differential modifications in organic acid production and flavonoids were reported in response to AM fungi with mutualistic versus parasitic phenotypes (Kaur et al. 2022). It is therefore possible that the observed shifts in the rhizosphere reflect the ability of this species to sustain beneficial interactions with AM fungi. However, our study included only one species that exhibited a distinct rhizosphere metabolome shift and strongly positive plant–soil feedback. Further validation with more species is needed to ascertain if there are consistent metabolic shifts across many species that exhibit positive PSF.

To date, we do not know if the ability to regulate AM symbiosis can be reliably related to known axes of variation in plant belowground functions. The ability to modify AM fungal community diversity and composition has been linked to root diameter (Ramana et al. 2023), which is an indicator of plant dependence on AM symbiosis (Ma et al. 2018; Bergmann et al. 2020); however, differential growth responses to different AM fungal species were not related to mycorrhizal dependency (Romero et al. 2023). Therefore, it seems that the ability of some species to maintain positive PSF via regulation of AM symbiosis may represent an axis of variation in plant functional space that is independent from other belowground axes described to date (Bergmann et al. 2020). Metabolomics may be a promising tool in describing this axis of variation and studying its consequences for vegetation dynamics.

## Author Contributions

M.S., M.M., K.K., and K.Z. conceptualised the study and organised experimental and laboratory work. G.H., S.S., and M.S. performed the experiment and carried out plant and soil measurements. M.S. and J.D. performed plant and soil data analysis. P.P. and S.P. carried out metabolomics measurements, data analysis, and interpretation. M.S. compiled the first draft of the manuscript, and all authors contributed to manuscript writing.

## Funding

This work was supported by Estonian Research Council grants PRG1065 (MM), PRG1836 (KK), PRG2584 (JD), IUT20‐31 (KZ), PRG1223 (KZ) and ETF9332 (MS). Estonian Ministry of Education and Research (Centre of Excellence AgroCropFuture, TK200, MS and MM). INRAE funding to MetaboHUB (ANR‐11‐INBS‐0010, PP and PS). INRAE funding to PHENOME (ANR‐11‐INBS‐0012, PP and PS). European Union (ERC grant PlantSoilAdapt, 101044424, MS).

## Conflicts of Interest

The authors declare no conflicts of interest.

## Supporting information


**Table S1:** Plant growth responses to soils conditioned by conspecifics versus other species (con/het contrast) and differential responses to soils conditioned by four different heterospecific neighbours (het contrasts).
**Table S2:** The effects of soil inoculation treatments (sieved inoculum versus whole soil inoculum) and species mean root colonisation rate by arbuscular mycorrhizal (AM) fungi on rhizosphere chemical composition.
**Table S3:** The effects of differences between conspecific and heterospecific soils in plant biomass and rhizosphere chemical properties at the end of soil conditioning phase on the outcome of plant–soil feedback (plant growth in conspecific versus heterospecific soil).
**Figure S1:** Experimental design.
**Figure S2:** Plant growth response to inoculation with whole soil versus sieved soil inoculum lacking arbuscular mycorrhizal (AM) fungi across ten temperate grassland species.
**Figure S3:** Untargeted metabolic profiles of rhizosphere soil solutions.
**Figure S4:** Predictive soil metabolomics of plant‐mycorrhizal interactions.

**Table S4:** The list and characteristics of metabolic markers forming five main clusters in the Ward clustering analysis of rhizosphere metabolomes of two plant species ( *Leontodon hispidus* and *Prunella vulgaris* ) in two soil inoculation treatments (whole or sieved inoculum).

**Table S5:** The list and characteristics of top metabolic predictors of root mycorrhizal colonisation percentage, presence/absence of root mycorrhizal colonisation and inoculation treatment (whole or sieved soil inoculum).

## Data Availability

All data and analysis code are openly available in the figshare repository: https://doi.org/10.6084/m9.figshare.29318639. Metabolomics data is available at Recherche Data Gouv: https://doi.org/10.57745/UP32DT.
