# Supplementary material for: Contrast in Mycorrhizal Associations Leads to Divergent Rhizosphere Metabolomes and Plant–Soil Feedback Among Grassland Species
Source: Ecol Lett. 2026 Feb 11;29(2):e70318. doi: 10.1111/ele.70318 (PMC12893406; doi:10.1111/ele.70318)
Supplement: Supplementary file 1 — Table S1: Plant growth responses to soils conditioned by conspecifics versus other species (con/het contrast) and differential responses to soils conditioned by four different heterospecific neighbours (het contrasts). Table S2: The effects of soil inoculation treatments (sieved inoculum versus whole soil inoculum) and species mean root colonisation rate by arbuscular mycorrhizal (AM) fungi on rhizosphere chemical composition. Table S3: The effects of differences between conspecific and heterospecific soils in plant biomass and rhizosphere chemical properties at the end of soil conditioning phase on the outcome of plant–soil feedback (plant growth in conspecific versus heterospecific soil). Figure S1: Experimental design. Figure S2: Plant growth response to inoculation with whole soil versus sieved soil inoculum lacking arbuscular mycorrhizal (AM) fungi across ten temperate grassland species. Figure S3: Untargeted metabolic profiles of rhizosphere soil solutions. Figure S4: Predictive soil metabolomics of plant‐mycorrhizal interactions. [file ELE-29-0-s003.pdf]

Supplementary methods

Mycorrhizal colonisation estimation

Dried roots were soaked in 2.5% KOH solution for 24 hours; then the solution was replaced and roots heated in a water bath at 90ºC for 5-10 minutes and rinsed thoroughly with tap water. In case of darker roots, additional bleaching with 30% hydrogen peroxide was performed at room temperature. The roots were rinsed and acidified in 4% HCl for 30 minutes to ensure attachment of the pigment to hyphae. Acidified roots were coloured with 0.05% trypan blue (in acidic glycerin solution) and heated at 90ºC for 10 minutes. To decolorize, the roots samples were incubated in acidic glycerin solution at room temperature for at least 24 hours. Root samples were placed on microscopy slides and observed at 400x magnification at 100 random locations along the roots to estimate the proportion of roots colonized by AM fungal structures (McGonigle et al. 1990).

Untargeted metabolomics

To assess the composition of organic compounds dissolved in the rhizosphere solution, leachate samples were resuspended in 100 µL MeOH:H_2O (70:30, v/v), sonicated for 2 min, then centrifuged (15 min, 4°C, 14000 *g*) before untargeted metabolomics was conducted using an ACQUITY ultra-high-performance liquid chromatograph (UHPLC) coupled to a SYNAPT G2Si quadrupole time-of-flight (Q-TOF) mass spectrometer equipped with an electrospray (ESI) source (Waters), as previously described in (Pétriacq *et al.*, 2017). The liquid chromatography–mass spectrometry (LCMS) analytical sequence included 68 unique biological samples (n = 7), 8 extraction blanks (prepared without leachate material and used to rule out potential contaminants detected by untargeted metabolomics), and 6 Quality Control (QC) samples that were prepared by mixing 20 µL from each sample. QC samples were used for *i*) the correction of signal drift during the analytical batch, and *ii*) the calculation of coefficients of variation for each metabolomic feature so only the most robust features are retained for chemometrics (Broadhurst *et al.*, 2018). Raw LCMS data were processed following the DIA MS2 deconvolution method using MS-DIAL software (v. 4.7; Tsugawa, Hiroshi *et al.*, 2015). Annotations were performed based on MS1 spectra and MS2 fragmentation information using the FragHUB database, including thousands of natural products (courtesy of Guillaume Marti, AgroMix MetaToul, France). Thus, putative annotation of differentially expressed metabolites resulted from MS-DIAL screening of the MS1 detected exact HR *m/z* and MS2 fragmentation patterns (Tsugawa, H. *et al.*, 2015). Additionally, the InChiKeys of annotated features were employed within ClassyFire to generate a structural ontology for chemical entities (Feunang *et al.*, 2016). Curation of metabolomic signals (CV QC < 50%, SN > 10) resulted in 1173 LCMS features, of which 547 remained unidentified, having no match with either MS1 or MS2, another 585 features suggested to be annotated metabolites, having a match with MS1 only (referred to as level 3 MS ID), and 41 features that were positively identified as metabolites, matching both MS1 and MS2 (referred to as level 2 MS ID).

Table S1. Plant growth responses to soils conditioned by conspecifics *versus* other species (con/het contrast) and differential responses to soils conditioned by four different heterospecific neighbours (het contrasts). Linear model results are shown for eight focal species sorted by their mean root AM fungal colonisation in the soil conditioning stage (AMF %, mean and standard error of the mean shown) when soils were inoculated with whole soil including arbuscular mycorrhizal (AM) fungi or sieved soil inoculum that excluded AM fungi (32 µm sieve). PSF - model coefficient showing ln-transformed biomass difference between the conspecific treatment and all heterospecific soil treatments, $R^2_{con/het}$ - variance in plant biomass explained by the contrast between conspecific and heterospecific soil; R^2_{het} - variance explained by the contrast between four heterospecific soils. Models significant at *p* < 0.05 are highlighted in bold.

		Sieved soil inoculum					Whole soil inoculum				
Species	AMF%	PSF	$R^2_{con/het}$	*P*	R^2_{het}	*P*	PSF	$R^2_{con/het}$	*P*	R^2_{het}	*P*
C. flacca	0.9 (0.7)	**-0.52**	**0.50**	**<0.001**	0.20	0.308	-0.26	0.10	0.130	0.09	0.701
F. rubra	6 (4.0)	**-0.77**	**0.22**	**0.018**	0.29	0.132	**-0.62**	**0.26**	**0.009**	0.25	0.200
S. vulgaris	16 (6.3)	**-0.23**	**0.24**	**0.012**	**0.40**	**0.040**	**-0.16**	**0.18**	**0.036**	0.22	0.261
B. media	18 (3.4)	**-0.56**	**0.18**	**0.038**	0.23	0.252	**-1.01**	**0.62**	**<0.001**	**0.47**	**0.021**
G. verum	25 (7.7)	**-1.14**	**0.21**	**0.027**	**0.51**	**0.012**	-0.23	0.01	0.692	**0.74**	**<0.001**
L. hispidus	40 (10.4)	0.01	<0.01	0.984	0.04	0.907	**1.74**	**0.42**	**<0.001**	**0.77**	**<0.001**
C. vulgaris	56 (7.2)	-0.12	<0.01	0.800	0.34	0.160	**1.41**	**0.27**	**0.022**	**0.89**	**<0.001**
P. vulgaris	59 (6.1)	-0.04	<0.01	0.957	**0.57**	**0.023**	-0.63	0.04	0.367	**0.78**	**<0.001**

Table S2. The effects of soil inoculation treatments (sieved inoculum *versus* whole soil inoculum) and species mean root colonisation rate by arbuscular mycorrhizal (AM) fungi on rhizosphere chemical composition. F- and P-values from linear mixed models including species as a random factor (n = 6 species; 7 replicates per species per treatment) are shown.
Variables were log-transformed if necessary to satisfy models assumptions.

		DOC (mg/L)		DOC (mg/L/g plant mass)		DON (mg/L)		Nitrate (mg/L)		Ammonium (mg/L)		Phosphate (mg/L)	
	Df	F	*P*	F	*P*	F	*P*	F	*P*	F	*P*	F	*P*
Inoculation (I)	1,76	0.98	0.324	2.01	0.160	6.46	**0.013**	0.07	0.798	8.13	**0.006**	3.03	0.086
AM fungal colonisation (C)	1,4	5.97	0.071	0.90	0.396	4.29	0.107	3.31	0.143	3.75	0.125	<0.01	0.999
I x C	1,76	57.6	**<0.001**	17.4	**<0.001**	27.1	**<0.001**	47.2	**<0.001**	5.70	**0.019**	1.53	0.200

Table S3. The effects of differences between conspecific and heterospecific soils in plant biomass and rhizosphere chemical properties at the end of soil conditioning phase on the outcome of plant-soil feedback (plant growth in conspecific *versus* heterospecific soil). Differences in plant biomass and rhizosphere chemistry and plant-soil feedback values were calculated as $\log_e$-transformed ratios of all pairwise comparisons between conspecific and heterospecific plant-soil replicates for each focal plant species. The results from linear mixed models are shown with plant-soil feedback (PSF) as the response variable, soil inoculation treatment and difference in a given predictor variable between con- and heterospecific soil and the interaction term as fixed factors, are shown. P-values for the main effect of difference and its interaction with inoculation treatment are shown, as well as the slopes of the relationship with PSF and its standard error (SE) for each inoculation treatment. Focal plant species identity and the species identity of conditioned soils were included in the models as random intercepts and inoculation treatment was included as a random slope(for allowing random variation in slopes between plant species). Slopes significantly deviating from zero (p < 0.05) are highlighted in bold.

	Whole inoculum		Sieved inoculum		Difference	Myc×Difference
Predictor	Slope	SE	Slope	SE	*P*	*P*
Plant dry mass (g)	0.061	0.064	**-0.088**	**0.028**	0.016	0.025
Dissolved organic C (mg/L)	0.282	0.393	0.139	0.264	0.420	0.758
Dissolved organic N (mg/L)	-0.453	0.241	0.235	0.168	0.674	0.028
Nitrate (mg/L)	0.131	0.175	-0.071	0.080	0.560	0.275
Ammonium (mg/L)	-0.138	0.418	0.461	0.326	0.365	0.258
Phosphate (mg/L)	0.060	0.116	**0.376**	**0.085**	0.000	0.027

Table S4. The list and characteristics of metabolic markers forming five main clusters in the Ward clustering analysis of rhizosphere metabolomes of two plant species (*Leontodon hispidus* and *Prunella vulgaris*) in two soil inoculation treatments (whole or sieved inoculum). The table is presented in a separate supplementary Excel file.

Table S5. The list and characteristics of top metabolic predictors of root mycorrhizal colonisation percentage, presence/absence of root mycorrhizal colonisation and inoculation treatment (whole or sieved soil inoculum). Specific and overlapping metabolic predictors for different mycorrhizal characteristics are shown. The table is presented in a separate supplementary Excel file.

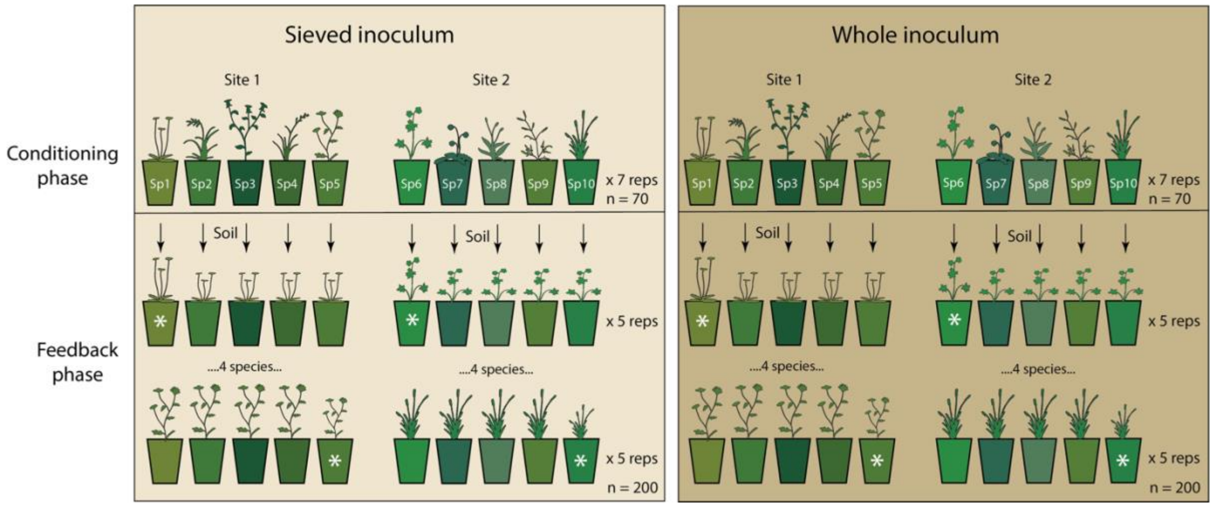

Figure S1. Experimental design. Ten grassland species from two sites were used as focal species. In the conditioning stage, each species was grown in monoculture in soil inoculated with either sieved inoculum that lacked AM fungi or whole soil inoculum, with seven replicates per soil treatment, resulting in a total of 140 pots. In the feedback stage, the conditioned soil from five out of seven replicates was each mixed with an equal weight of sterilised background soil mixture; soil from each pot was kept separate to maintain true replication and was divided into five smaller pots to be used as a growth substrate for seedlings of five species from the same site. Due to poor germination, four species per site were grown as focal species in the feedback stage instead of planned five. Each focal species was subjected to five soils: conspecific soil (shown with an asterisk in the figure) and four soils conditioned by other species from the same site, with each soil represented with five independent replicates. As the same setup was repeated for sieved and whole inoculum treatments, this resulted in a total of 400 pots (8 focal species x 5 soil identities x 5 replicates x 2 inoculation treatments). Plant-soil feedback (PSF) was calculated as a log-ratio of plant growth in conspecific soil vs a given heterospecific soil. PSF is positive for species in the top row of feedback stage (plants are larger when grown on conspecific than heterospecific soil) and negative for species depicted in the bottom row.

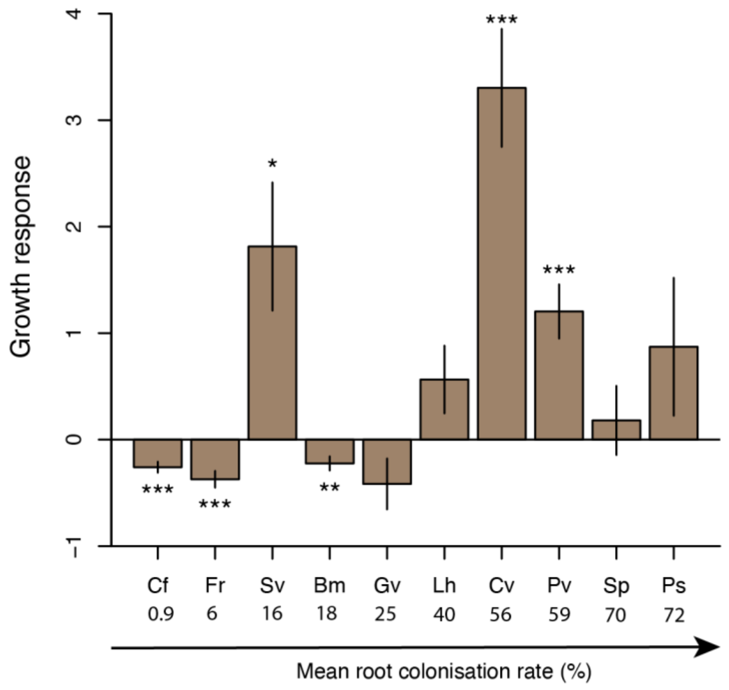

Figure S2. Plant growth response to inoculation with whole soil *versus* sieved soil inoculum lacking arbuscular mycorrhizal (AM) fungi across ten temperate grassland species. Growth response was estimated from a linear model with the soil inoculation treatment as a fixed factor and ln-transfromed total dry mass as the response variable. Positive and negative values indicate better or worse plant growth with the whole than sieved inoculum, respectively. N=7 per treatment per species. Model coefficients, standard errors and their significance of deviation from zero based on a t-test are shown. * $p < 0.05$; ** $p < 0.01$; *** $p < 0.001$. Mean AM fungal root colonisation rate (%) is indicated for each species at the bottom of the figure. *Cf – Carex flacca; Cv – Carlina vulgaris; Bm – Briza media; Fr – Festuca rubra; Gv – Galium verum; Lh – Leontodon hispidus; Ps – Pimpinella saxifraga; Pv – Prunella vulgaris; Sv – Silene vulgaris; Sp – Succisa pratensis*

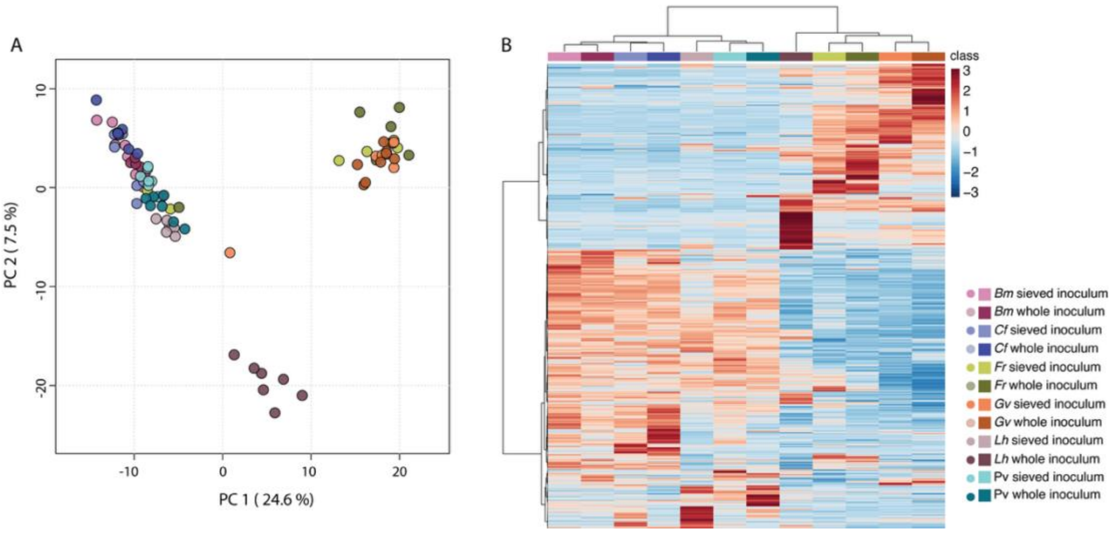

Figure S3. Untargeted metabolic profiles of rhizosphere soil solutions. (A) Principal Component analysis (PCA) of 1173 metabolomic features using normalised intensities (median normalisation, cube root transformation and Pareto scaling). (B) Heatmap clustering analysis (Pearson's correlation, Ward clustering) of 668 rhizochemical markers showing significant, contrasting changes in response to species and the presence of arbuscular mycorrhizal (AM) fungi. All markers presented statistically significant variations (ANOVA, $p < 0.01$ with adjusted FDR correction). Each column represents the conditions for species with/without AM fungi (n=7). Each row indicates the significant metabolic markers with relative intensity shown as a heatmap (blue, depleted; red, accumulated). *Cf – Carex flacca; Bm – Briza media; Fr – Festuca rubra; Gv – Galium verum; Lh – Leontodon hispidus; Pv – Prunella vulgaris*

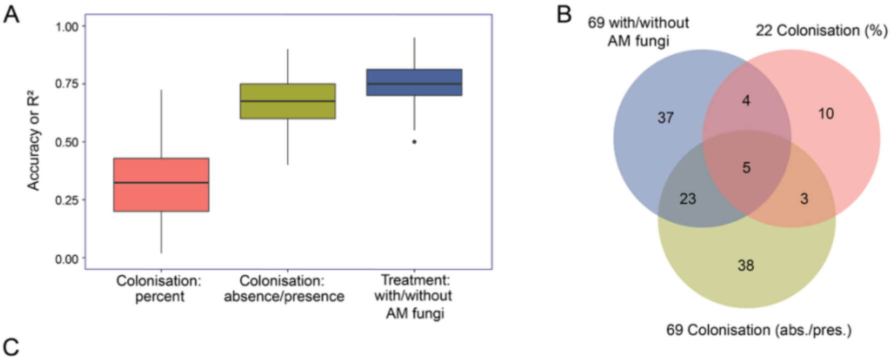

C

	Specific			Overlapping			
Metabolic pathways	Treatment: with/without AM fungi	Colonisation (%)	Colonisation (abs./pres.)	Treatment vs Colonisation (abs./pres.)	Treatment vs Colonisation (%)	Colonisation (abs./pres.) vs Colonisation (%)	Common
Alcohols and polyols	0	0	0	0	0	0	0
Alkaloids	0	0	1	0	0	0	0
Amino acids and derivatives	0	0	1	1	0	0	1
Benzenoids	3	0	3	0	0	0	0
Carbohydrates and derivatives	1	0	0	0	0	0	0
Hydrocarbon derivatives	0	0	1	0	1	0	0
Indoles	0	0	0	0	0	0	0
Lignans	0	0	0	0	0	0	0
Lipids and derivatives	2	1	3	1	0	0	1
Nucleotides and derivatives	0	0	0	0	0	0	0
Organic acids and derivatives	1	0	4	2	0	0	0
Organoheterocyclic compounds	5	3	5	2	0	1	0
Phenylpropanoids and polyketides	3	1	5	2	1	0	2
Terpenes	2	1	2	3	0	1	0
# Total annotated markers	17	6	25	14	2	2	4
**# Total markers**	**37**	**10**	**38**	**23**	**4**	**3**	**5**

Figure S4. Predictive soil metabolomics of plant-mycorrhizal interactions. (A) Accuracy of predicting mycorrhizal characteristics based on the rhizosphere metabolome. Y-axis shows model accuracy calculated as the proportion of correctly predicted classes (or R^2 for the extent of mycorrhizal colonisation as a continuous variable) in 100 general linear model fits between predicted and observed values. Predicted values were calculated based on the rhizosphere metabolome. Predicted mycorrhizal characteristics included root mycorrhizal colonisation percentage as a continuous variable and the presence/absence of root mycorrhizal colonisation and inoculation treatment (whole or sieved soil inoculum excluding AM fungi) as categorical variables. (B) Venn diagram showing the numbers of specific, overlapping and common metabolic predictors for different mycorrhizal characteristics among the top predictors defined as those selected in >60% of predictive models. (C) Distribution of the top predictors among the metabolic pathways based on chemical ontologies.
